# Competition among cities for export trade brings diversification: The experience of China’s urban export trade development

**DOI:** 10.1371/journal.pone.0271239

**Published:** 2022-09-15

**Authors:** Enkang Li, Yingyi Ma, Yi Wang, Yu Chen, Bo Niu

**Affiliations:** 1 School of Architectural Engineering, Jinling Institute of Technology, Nanjing, Jiangsu, China; 2 School of Economics and Management, Nanjing University of Science and Technology, Nanjing, Jiangsu, China; 3 School of Geography, Nanjing Normal University, Nanjing, Jiangsu, China; Shandong University of Science and Technology, CHINA

## Abstract

Market competition is considered to have a significant impact on product diversification, but related issues are rarely discussed on a city scale. To analyze the diversification of export commodities and export market of 270 Chinese cities, this study uses data from 2000 to 2017 based on the commodity concentration index, market concentration index, export similarity index, spatial stratified heterogeneity, and the Almon lag model. The study’s findings are: (1) The diversification of exports in most Chinese cities increased, which was higher in more developed cities in the southeast than in less developed ones in the northwest. With time, the degree of commodity and market diversification in some developed southeast Chinese cities (e.g., Shanghai) declined. This indicates the difference in and complexity of the evolution of export development in hundreds of Chinese cities between 2000 and 2017. (2) The export competition between cities became increasingly fierce, which effectively urged most of them to improve their export diversification levels. Facing increasing export competition pressure, 81.11% of the total number of cities will enhance the diversification of export commodities to cope with challenges posed by other cities. But only 56.67% will further expand the export market when the pressure of export competition increases. The biggest contribution of this study is the finding that for most Chinese cities, when export competition from other domestic cities increases, increasing diversification of products becomes a wise choice. However, the influence of competition on the diversification degree of the city’s export market is relatively weak. This study provides not only a new perspective for existing research on urban export trade, but also valuable information for cities to form a more profitable and robust trading system.

## Introduction

Since joining the World Trade Organization (WTO), China’s trade has developed at an amazing speed [1, 2]. It has become the *world’s factory* [3], providing rich raw materials and commodities to other countries, and being a strong link in the global value chain (GVC) [4]. This development is closely related to the dynamic growth of export trade in hundreds of Chinese cities, especially in eastern coastal areas. Because cities have varying natural resources and geographical locations, their export commodities have different structures and flow to various markets. For example, a high proportion of exports from Dalian City and Qingdao City in North China flows to Japan and South Korea [5], and from Shenzhen City and Guangzhou City in South China to Hong Kong [6, 7].

Existing studies on the export trade of Chinese cities have mainly focused on two aspects. One is the impact of exports on urban development, including economic growth [8, 9], urbanization [10], issues around left-behind children [11], urban residents’ happiness [12], urban pollution, and environmental protection [13, 14]. The other aspect is related to factors affecting a city’s export trade, which includes environmental regulations [15], financial development and constraints [16], and air pollution [17].

A few studies have examined the diversification of urban export products and commodity, and the destination market structures of the export trade of Chinese cities. The continuous adjustments in the pace of China’s economic transformation should advance the optimization of urban export trade that eliminates dependence on labor-intensive industries. Improving the diversification of commodities and actively exploring overseas markets are important measures for Chinese cities to enhance their export levels. China has many cities, among which export products overlap and compete widely. Studies have shown that the increase of export competitive pressure will affect the change of export product types and combinations [18, 19]. However, whether this kind of competition affects the commodity and market diversification of urban exports has not been addressed by relevant studies. Therefore, this study focuses on the following scientific question: as an “economic man” with a natural desire for economic growth, how does a city react in terms of commodity diversification and market diversification when it competes with other cities in export trade?

**Fig 1:**
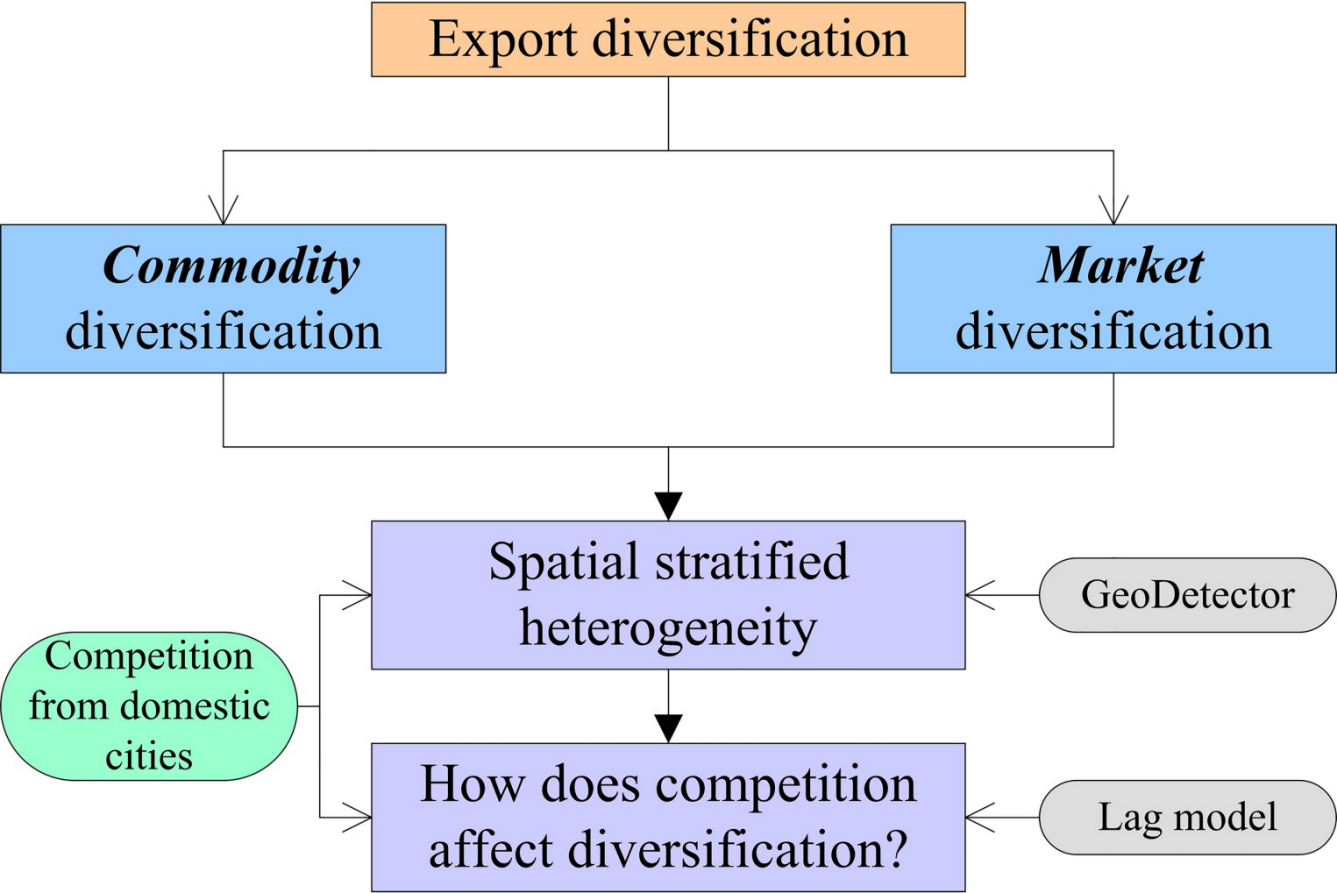
Framework.

The framework of this study is divided into two parts (Fig 1). First, we calculated the degree of commodity and market diversification of Chinese cities’ exports to the world. Second, we calculated the pressure of export competition faced by each Chinese city and then analyzed how it affects export diversification. From the urban scale, this paper analyzes how different regions will change their export structure and destination when facing the pressure of export competition. The contribution of this research lies in the quantitative measurement of the different responses of Chinese cities to export competition pressure using the econometric model.

### Literature review

Cities are prominent worldwide because of the concentration of social and economic activities within them [20]. Research on export trade based on the analysis of countries has begun to turn its focus to cities. As Kalafsky argued, export dynamics should be viewed increasingly from an urban frame [21]. One of the pillars of the framework of trade research at the urban scale is the in-depth analysis of the factors affecting urban export.

The improvement of transportation infrastructure will undoubtedly bring opportunities for rapid development of a city’s export [22]. The construction of expressways can effectively promote the export volume of a city. For border cities, there is a closer relationship between export-led growth and transportation accessibility [23]. Contrastingly, the level of innovation [24], the stability of the financial system [25], the environmental policies made by the government [15], and the tax incentives provided to enterprises [26] will have a great impact on the export development of a city. There are also occasional events that can affect a city’s exports, such as a financial crisis [27] and health-related emergencies such as the COVID-19 pandemic [28].

Despite the remarkable findings of the above studies, when discussing the different factors affecting a city’s exports, researchers have mostly focused on how the *total value* of the city’s exports was affected. Total value is indeed one of the most important indicators in export trade research, and it is very easy to obtain such data. However, there is another very important aspect of export trade analysis that has received little attention: the discussion of the structure of exports and the spatial distribution of the destination countries receiving a city’s exports. In fact, most studies on export product structure and destination distribution are based on the *country*. Many scholars have discussed the level of export diversification [29–33] and what factors lead to diversification [34–37]. However, export diversification at the urban scale is still scarce, and only a few scholars such as He et al. have discussed relevant issues [38–40].

Another point that must be considered is that research on urban export trade is still lacking from the perspective of mutual *competition*. The reason why different cities compete in export trade is that the growth of export trade can effectively promote urbanization [41, 42] and urban economic development [43, 44]. Although over-reliance on exports can also lead to environmental damage [45, 46], the benefits of an export-oriented economic model have been encouraging an increasing number of cities to join the race. In China, some neighboring coastal cities are dotted with factories making identical or similar products to compete in the world market. Given the above, does this competition between domestic cities influence each city’s decisions on product mix and destination market selection? To the best of our knowledge, no previous studies have provided an answer to this question, and this is the research gap that this study tries to fill.

## Methodology

### Methods

#### Commodity and market concentration indices

Typically, the Gini-Hirschman coefficient [47] is applied to calculate the commodity concentration index (CCI) and market concentration index (MCI) as follows [48–50]:

$$CCI_{i}=100\times {\left(\sum_{c}{\left(\frac{\exp_{ic}}{\exp_{i}}\right)}^{2}\right)}^{0.5}$$ (1)

where exp_*ic*_ is the commodity *c* value in city *i*‘s global exports, and exp_*i*_ is city *i*‘s total export value in the world market. The CCI value is between 0 and 100. The larger the value, the lower the degree of commodity diversification; the smaller the value, the higher the degree of commodity diversification.

$$MCI_{i}=100\times {\left(\sum_{m}{\left(\frac{\exp_{im}}{\exp_{i}}\right)}^{2}\right)}^{0.5}$$ (2)

where exp_*im*_ is city *i*‘s export value to country *m*, and exp_*i*_ is city *i*‘s total export value in the world market. The MCI value is between 0 and 100. The larger the value, the lower the degree of market diversification; the smaller the value, the higher the degree of market diversification.

#### Export similarity index

As a reflection of the similarity in exports between regions, the export similarity index (ESI) has been widely adopted by scholars since its inception, especially those focusing on economic geography. The ESI has expounded many academic achievements involving international trade, industrial economies, and other multidisciplinary fields [51–53]. It can indicate the degree of convergence of commodities in two regions in a certain market. The higher the ESI, the more intense the competitive relationship is [54, 55]. The formula is as follows:

$$ESI_{ijk}=100\times \sum_{l}\min(\frac{\exp^{l}{}_{ik}}{\exp_{ik}},\frac{\exp^{l}{}_{jk}}{\exp_{jk}})$$ (3)

where *EZI*_*ijk*_ is the similarity between cities *i* and *j* regarding exports in the market *k*; $\frac{\exp^{l}{}_{ik}}{\exp_{ik}}$ represents the ratio of commodity *l* that is exported by city *i* to market *k* to its total exports to market *k*; $\frac{\exp^{l}{}_{jk}}{\exp_{jk}}$ represents the ratio of commodity *l* that is exported by city *j* to market *k* to its total exports to market *k*. The value of *ESI*_*ijk*_ is between 0 and 100. The closer it is to 100, the more similar the export products of cities *i* and *j* in market *k*. In short, a high *ESI*_*ijk*_ indicates high export competition between cities *i* and *j*; a low *ESI*_*ijk*_ indicates less intense export competition between cities *i* and *j*. In this study, the market *k* is the global market.

Based on the ESI, this study creates the average export similarity index (AESI) as an indicator that reflects the competitive pressure that each city faces from other cities. The formula is as follows:

$$AESI_{i}=\frac{\sum_{j}ESI_{ijk}}{269}$$ (4)

This study’s research subject includes 270 cities, where each city has one ESI with every other city. This means that each city has 269 ESIs. The higher the *AESI*_*i*_, the greater the competitive pressure city *i* faces from other cities in the global market.

Geographical detector

Geographical Detector (Geodetector), is a statistical tool that measures Spatial Stratized Heteroeity (SSH). In this study, we use this method to analyze the extent to which the independent variable X (AESI) explains the spatial differentiation of the dependent variable Y (CCI and MCI). The calculation formula is as follows [56]:

$$q=1-\frac{\sum_{h=1}^{L}N_{h}\sigma_{h}^{2}}{N\sigma^{2}}$$ (5)

*q* represents the explanatory power of *X* (AESI) on *Y* (CCI and MCI), and the larger the *q*, the stronger the explanatory power; *q*∈[0,1]. *h* represents the stratification of independent variables (the independent variable AESI is divided into 5 levels using quantile classification according to the study of Ding et al. [57]). *N*_*h*_ and *N* represent the number of units in layer *h* and whole region, respectively. $\sigma_{h}^{2}$ and *σ*^2^ are the variances of Y in layer *h* and whole region, respectively.

The Almon lag model (ALM)

There is a lag in the influence of competitive pressure (AESI) on city export diversification (CCI and MCI). This is because the export enterprises in a city experience a change in competitive pressure first and then adjust the structure of export products. This process requires time. In this study, the distributed lag model is used to analyze the influence of AESI on CCI and MCI in the current and lag periods. The finite distributed lag model can be presented as follows [58]:

$$Y_{t}=a+\sum_{v=0}^{s}b_{v}X_{t-v}+\mu_{t}$$ (6)

where *Y*_*t*_ is the variable to be studied (i.e., CCI and MCI); *a* is the free term; *b*_*v*_ is the sum of the values of the pulse response function; *X* is the explanatory factor (i.e., AESI); *μ*_*t*_ is white noise; *t* is time; *v* is the lag period; and *s* is the number of lag periods. This model describes how the lagged effects of changes in *X* are distributed over time [59].

To reduce the impact of multicollinearity, the Almon lag model (ALM) is used in this research [60, 61]. The Almon polynomial transformation is expressed as follows:

$$b_{\varphi }=a_{0}+a_{1}\varphi +a_{2}\varphi^{2}+\cdots +a_{m}\varphi^{m},(m<v)$$ (7)

where *φ* is low order polynomial; and *m* represents lag weights.

### Data

The export data for each city are obtained from the customs database of China which is a database that many economists use [62–65] (service trade data are not included). Based on the enterprise codes, the export situation of enterprises in the same cities is summarized and used as the original export data of those cities. In addition, the customs database includes a large span of data collection years, which has resulted in multiple versions: HS1996, HS2002, HS2007, HS2012, and HS2017. Although the differences between the versions are not significant from a macro viewpoint, each version has a certain degree of fine-tuning when compared with its previous version. Hence, they must be recoded systematically. According to the comparison table between the different commodity codes issued by the United Nations Trade Database (https://unstats.un.org/unsd/trade/classifications/correspondence-tables.asp), this study uniformly adjusts the eight-digit commodity codes of every year of the customs data to the HS1996 version with six-digit codes, so that the CCI, MCI, and AESI between the different years can be measured comparatively. Based on data availability, 270 cities in mainland China are selected.

### Study area

**Fig 2:**
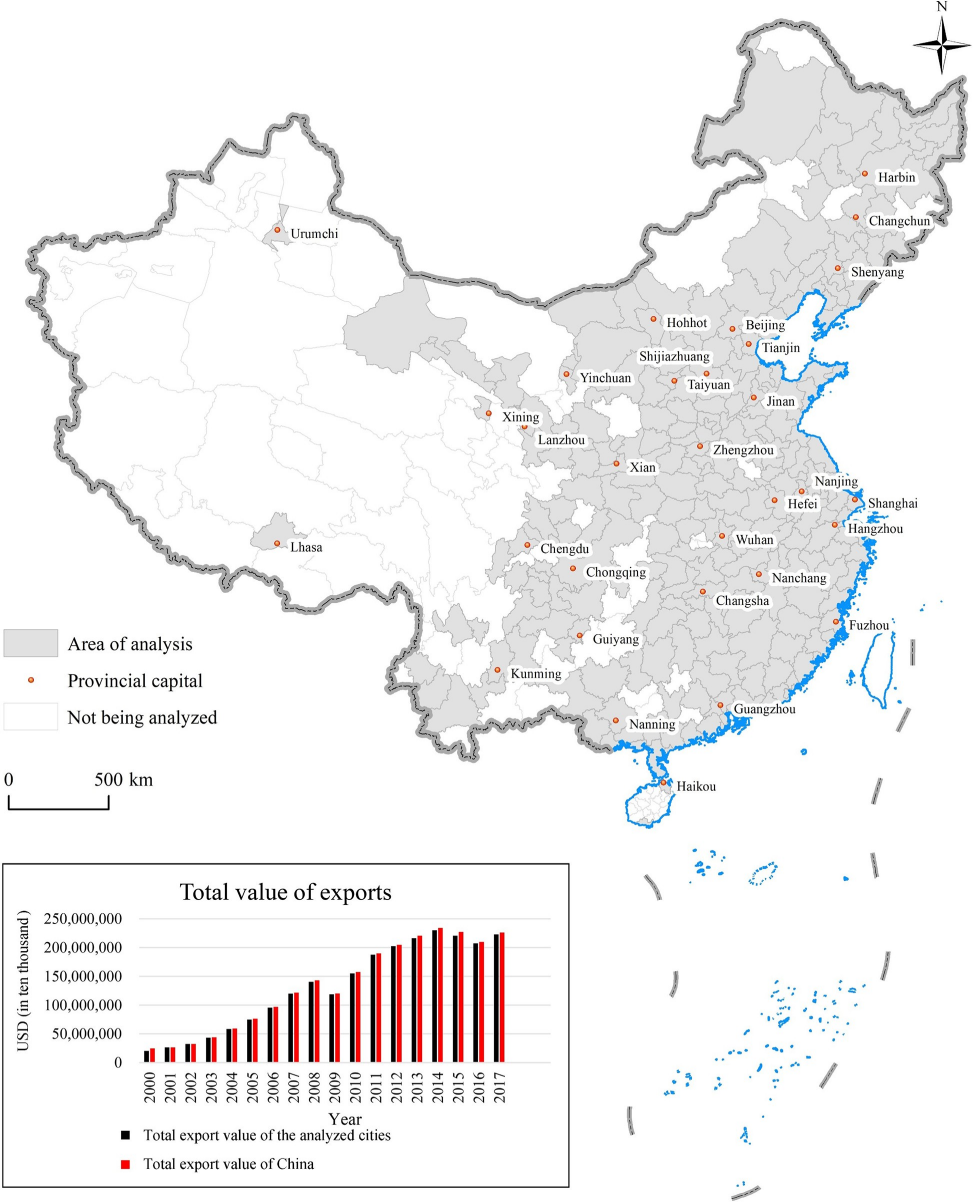
Study area.

As shown in Fig 2, the cities analyzed are mainly located in the central and eastern regions of China. Moreover, the total export value of the 270 cities from 2000 to 2017 is close to that of the country. Therefore, the selection of these cities for analysis can comprehensively reflect the development characteristics of China’s urban exports.

## Results

### The spatiotemporal pattern of export diversification

#### Commodity diversification

**Fig 3:**
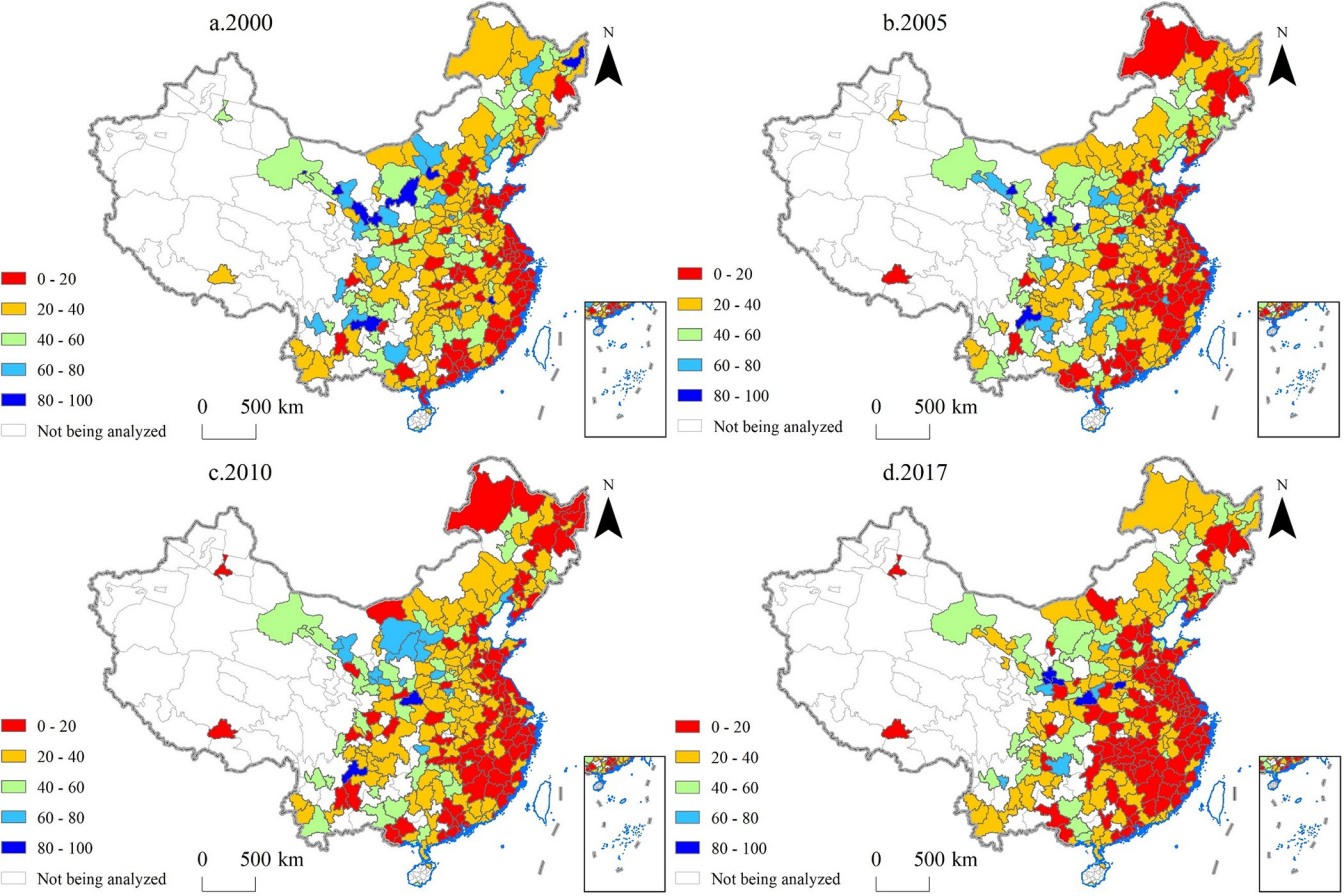
Commodity concentration index (CCI) of the 270 Chinese cities analyzed in the study in the years 2000, 2005, 2010 and 2017.

From 2000 to 2017, an increasing number of Chinese cities diversified their export commodities. In 2000, only 73 cities had a CCI of less than 20, but the number increased to 117 in 2017 (Fig 3). The decline in CCI was significant in some cities. For example, Changchun City (the capital of Jilin Province) and Urumqi City (the capital of Xinjiang Province) both saw their CCI drop by more than 70% between 2000 and 2017.

However, some cities, especially those with higher economic levels, saw a rise in CCI. This means that exports from these cities became less diversified. The CCI of more developed cities, including Zhengzhou City (the capital of Henan Province), Chengdu City (the capital of Sichuan Province), and Shanghai City (a municipality directly under the central government), increased by more than 100% between 2000 and 2017.

### Market diversification

**Fig 4:**
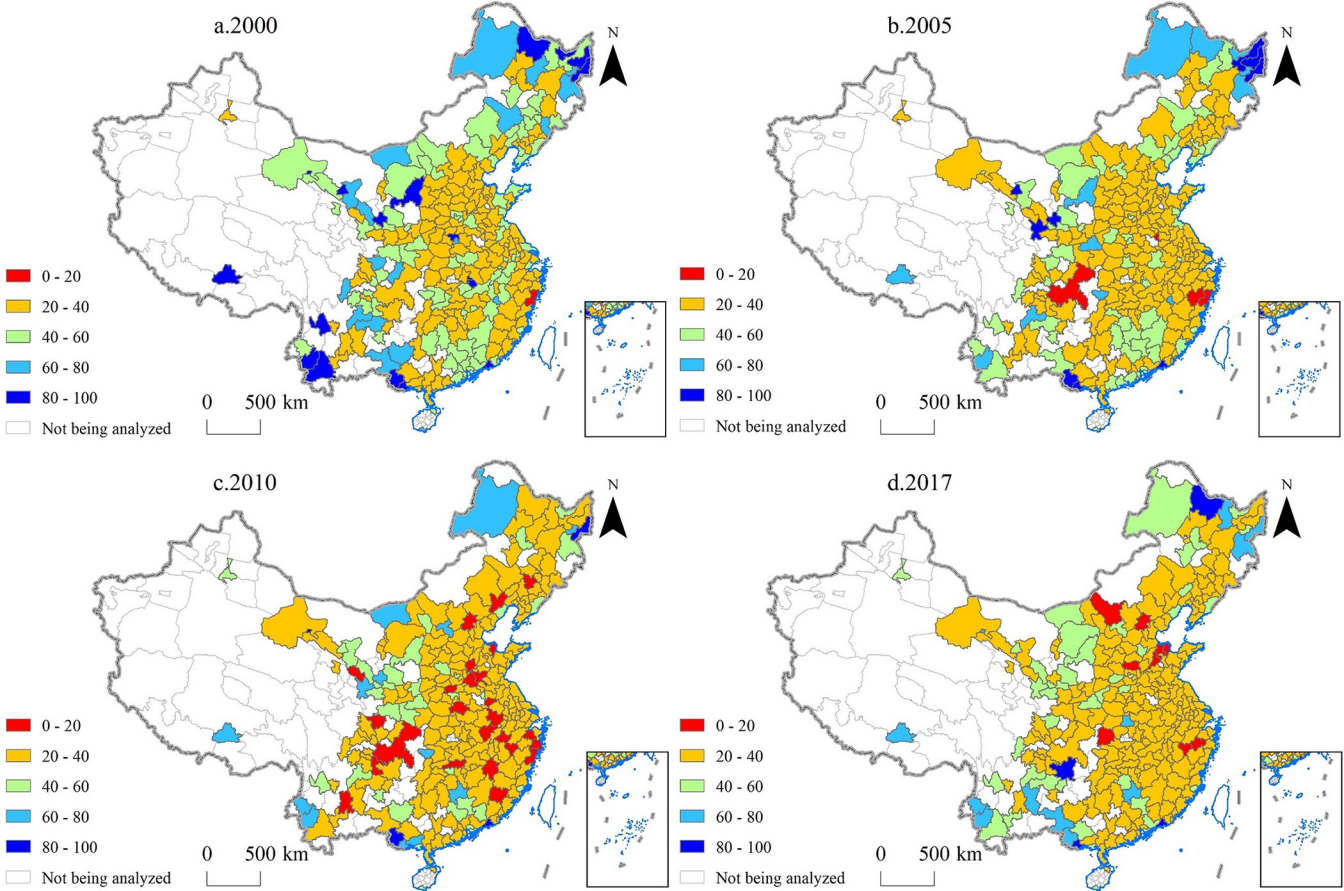
Market concentration index (MCI) of the 270 Chinese cities analyzed in the study in the years 2000, 2005, 2010 and 2017.

As can be seen in Fig 4, the MCI of most cities in China was between 20 and 40 in 144 (year 2000), 182 (2005), 192 (2010), and 204 (2017) cities. The increase in the number of cities in the MCI range of 20–40 also reflects the increasing diversification of China’s urban export markets. In 2017, the average MCI for 270 cities was 33.46, which was 23.40% lower than the average in 2000.

Unlike the CCI, the number of cities with MCI below 20 was relatively small. For example, in 2017, only nine cities, including Jinan City (the capital of Shandong Province) and Jinhua (in Zhejiang Province), had an MCI less than 20. In 2010, although the MCI of many cities was below 20, the distribution was dispersed, and there was no spatial feature of clustering distribution like the CCI.

Most cities’ export markets became more diversified, but some did not. Of the 270 cities, 51, including Xi’an City (the capital of Shanxi Province) and Wuhan City (the capital of Hubei Province), showed a trend of growth in MCI from 2000 to 2017. In other words, their export markets were less diverse. For example, 14.49% and 8.64% of Xi’an City’s total export value in 2000 were exported to the United States and Hong Kong, respectively. In 2017, these figures increased to 17.35% and 26.99%, respectively.

As can be seen, the spatial difference of MCI is smaller than that of CCI. This is because a city can choose from thousands of commodities to export (the mix of exports varies greatly from city to city) but will have only a hundred or so markets to choose from. Taking 2017 as an example, 45.56% of cities chose the United States as their largest export market, while 14.81% and 8.52% chose Hong Kong and South Korea, respectively, as their largest export destinations (see Appendix A in S1 Appendix).

### The impact of competition on export diversification

#### The export competition that each city faces

**Fig 5:**
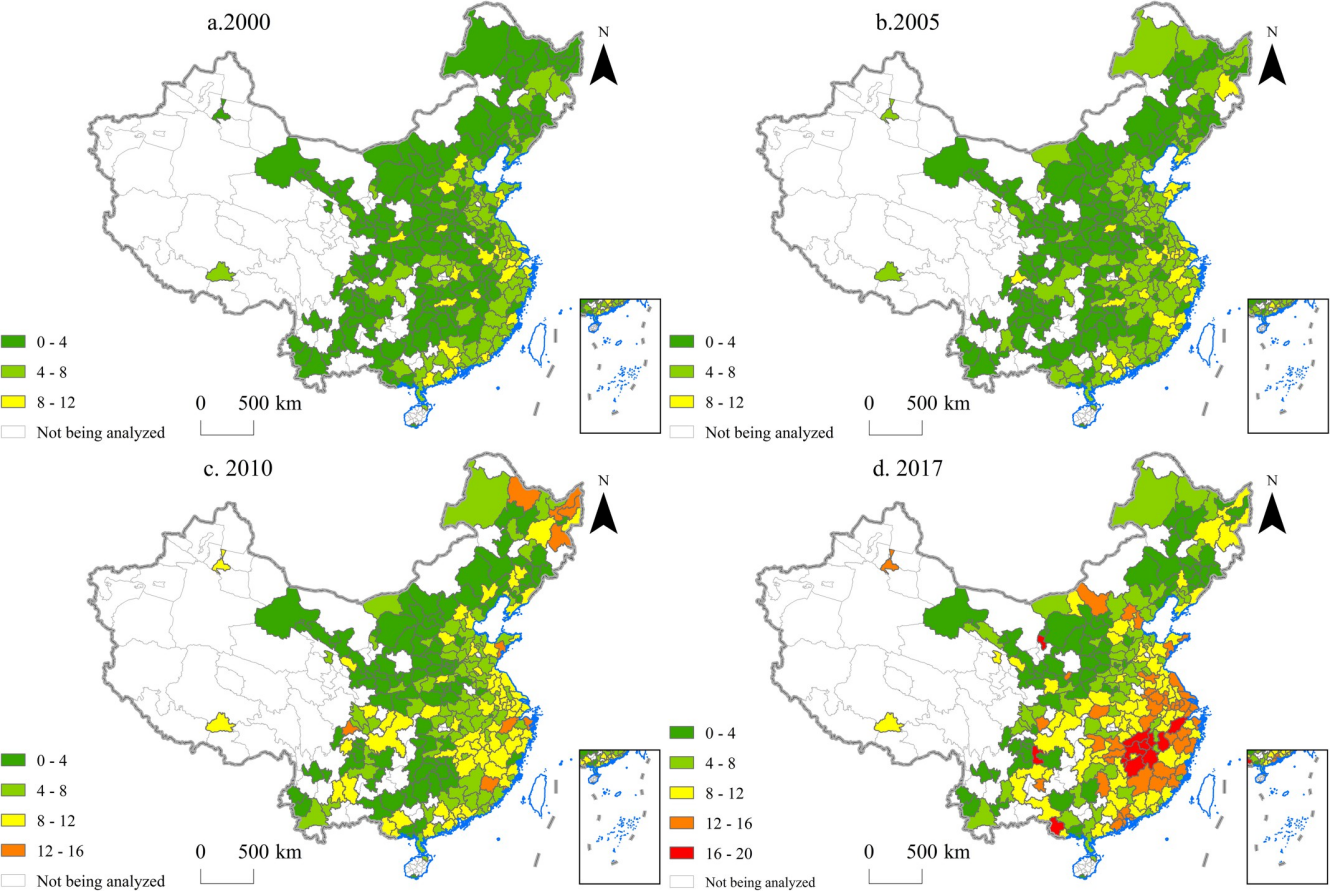
Average export similarity index (AESI) of the 270 Chinese cities analyzed in this study.

As shown in Fig 5, cities in eastern and central China had significantly higher AESI values than those in the west. For example, in 2000, seven provincial capitals in the west and three in the east had an AESI of less than 5 and greater than 10, respectively. In 2017, the pattern changed slightly as many cities in the Jiangxi Province had significantly higher AESI values than their surrounding cities. In 2017, 11 of the 270 cities had an AESI greater than 16, of which 7 were in the Jiangxi Province (Fig 5). The higher AESI in Jiangxi is due to the intense competition among cities in the province (see Appendix B in S1 Appendix). The time variation of the AESI was also significant. In 2000, most cities had an AESI of less than 8. However, in 2017, 49.63% of the cities had an AESI greater than 8 (Fig 5). This change reflects the growing export competition among Chinese cities.

### Spatial stratified heterogeneity: Spatial interaction of competition and diversification

**Fig 6:**
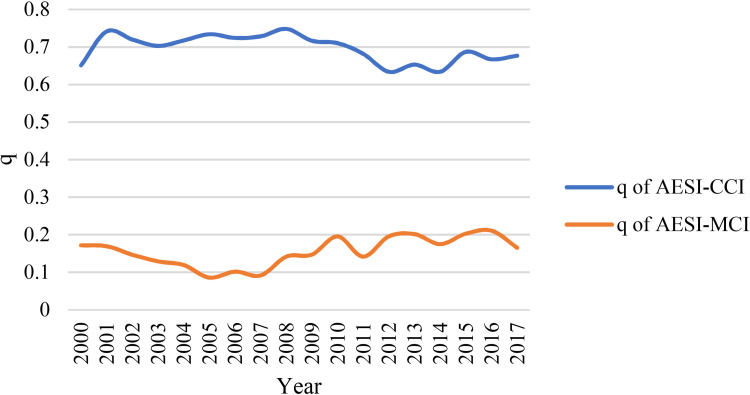
q of AESI-CCI and AESI-MCI (p-value is less than 0.01 every year).

We calculated the annual spatial interaction, which is the q value in the figure below (Fig 6). It is clear that AESI has a stronger explanatory power to the spatial pattern of CCI, and its q value remains in the range of 0.6–0.8 for a long time. In contrast, AESI is relatively weak in explaining the spatial pattern of MCI. q of AESI-MCI remained in the range of 0.1–0.2 for a long time.

However, this is only a preliminary discussion of the relationship between AESI and two diversity indicators from a spatial analysis perspective. To what extent do changes in AESI affect CCI and MCI? The effect of AESI on the spatial pattern of MCI is weak from the perspective of spatial interaction, but does this mean that changes in the AESI of a city do not affect its MCI? These will be of the greatest concern to us.

### The effect: Competition brings diversification

Both the AESI and CCI were panel data (18 years, 270 cities). Using Stata/MP 16.0, we found that AESI and CCI passed the panel unit root test (PURT) [66] and panel co-integration test (PCIT) [67] (Table 1). In the panel Granger causality test (PGCT) [68], we found that AESI was the Granger cause of CCI. Because under all three criteria (AIC: Akaike’s Information Criterion; BIC: Bayesian Information Criterion; HQIC: Hannan and Quinn’s Information Criterion) [69–71], the null hypothesis H_a_ (AESI is not the Granger reason for CCI) can be significantly rejected (the p-value of both Z-bar and Z-bar tilde is below 0.01) (Table 2). Similarly, we found that CCI is also the Granger cause of AESI. In other words, AESI and CCI are bi-directional causality. Thus, AESI and CCI are endogenous. In the face of endogeneity, one of the common ways to solve the problem is by using *lag variables*. Therefore, the ALM was used to analyze how a change in AESI affects CCI.

**Table 1:** Results of panel unit root test (PURT) and panel co-integration test (PCIT) of CCI and AESI.

Panel unit root test				Panel co-integration test
Variable	Adjusted t	Number of panels	Number of periods	Variance ratio
CCI	-14.5220***	270	18	-6.4307***
AESI	-5.4053***	270	18	

Note:

*: p < 0.1

**: p < 0.05

***: p < 0.01.

**Table 2:** Results of panel Granger causality test (PGCT) between average export similarity index (AESI) and commodity concentration index (CCI).

Criterion	Items	Optimal lag period	H_a_: AESI is not the Granger reason for CCI	Optimal lag period	H_b_: CCI is not the Granger reason for AESI
AIC	W-bar	4	8.8011	4	8.1628
	Z-bar		27.8922***		24.1839***
	Z-bar tilde		2.8121***		1.9712**
BIC	W-bar	1	2.5249	1	1.8316
	Z-bar		17.7179***		9.6627***
	Z-bar tilde		11.8639***		5.8083***
HQIC	W-bar	4	8.8011	4	8.1628
	Z-bar		27.8922***		24.1839***
	Z-bar tilde		2.8121***		1.9712**

Note:

*: p < 0.1

**: p < 0.05

***: p < 0.01. W-bar = average Wald statistic; Z-bar = standardized average Wald statistic ($\bar{Z}$); Z-bar tilde = standardized average statistic ($\tilde{Z}$). Owing to the development of measurement methods on Stata/MP 16.0, this study was able to determine the optimal lag by using the *xtgcause* command.

**Fig 7:**
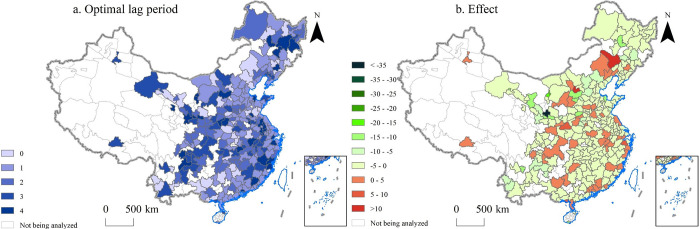
The optimal lag period and effect of average export similarity index (AESI) on commodity concentration index (CCI). The optimal lag period is the lag period with the minimum mean standard error. Meaning of effect: If AESI is increased by *one* unit, CCI will increase/decrease by the corresponding amount. CCI’s increases are expressed as positive values and decreases as negative values.

As shown in Fig 7A, the optimal lag period obtained by most cities is greater than 1 when analyzing the effect of AESI on CCI. For most Chinese cities, an increase in AESI led to a decrease in CCI (Fig 7B). In other words, the more a city faces competitive pressures in export, the more it diversifies its export commodities. This shows that the city is also a *rational economic man* to some extent. Increasing commodity diversification can prevent trade risks caused by commodity concentration and builds a healthier and stronger export system. China’s export trade has developed rapidly in the more than ten years since China’s accession to the WTO. In the face of the vast world market, although Chinese cities are competitive with each other, the huge scale of the destination market still makes it one of the important development paths for foreign trade enterprises in most cities to increase product types that will help obtain profits and cope with competition [72].

However, 18.89% of the cities registered a positive effect of AESI on CCI (orange and red in Fig 7B). An increase in AESI would result in an increase in CCI. For example, the impact of AESI on CCI in Chongqing City, Xi’an City, Fuzhou City was 2.04, 1.37, and 1.14, respectively. Let us take Chongqing as an example to discuss why competition leads to reduced product diversification in some cities. Since 2010, in the face of fierce export competition from domestic cities, Chongqing’s foreign trade policy and economic development path have undergone major adjustments. In the same year, Chongqing has brought electronics giants to the city, including HEWLETT-PACKARD, Acer, and Foxconn. The original relatively dispersed export commodity structure has rapidly changed to electronic products as the leading export structure. In 2017, 45.79% of the total value of Chongqing’s exports were electronic products. Market subjects respond to increased competition by dropping their worst performing products [73]. For each city, although it faces competition from other cities in China, the path chosen is different. This largely depends on the city’s geographical location, policy advantages, and economic basis. Chongqing is one of the four municipalities directly under the central Government in China, and also a window of western China’s opening to the outside world, to which the Chinese central government attaches great importance. Therefore, for Chongqing, there are certain advantages to introduce the world’s high-tech companies to set up factories there and produce leading products for export.

Both AESI and MCI were panel data. Using Stata/MP 16.0, we found that AESI and MCI passed the PURT and PCIT (Table 3). In the PGCT, we found that AESI was the Granger cause of MCI (Table 4, column 4). Similarly, we found that MCI is also the Granger cause of AESI. In other words, AESI and MCI are bi-directional causality. Thus, AESI and MCI are endogenous. As in the previous analysis, ALM was used to calculate the effect of AESI on MCI.

**Table 3:** Results of panel unit root test (PURT) and panel co-integration test (PCIT) of MCI and AESI.

Panel unit root test				Panel co-integration test
Variable	Adjusted t	Number of panels	Number of periods	Variance ratio
MCI	-16.1943***	270	18	-6.0628***
AESI	-5.4053***	270	18	

Note:

*: p < 0.1

**: p < 0.05

***: p < 0.01.

**Table 4:** Results of the panel Granger causality test (PGCT) between average export similarity index (AESI) and market concentration index (MCI).

Criterion	Items	Optimal lag period	H_a_: AESI is not the Granger reason for MCI	Optimal lag period	H_b_: MCI is not the Granger reason for AESI
AIC	W-bar	4	7.4847	4	8.2290
	Z-bar		20.2444***		24.5684***
	Z-bar tilde		1.0778		2.0584**
BIC	W-bar	1	1.7422	1	2.5360
	Z-bar		8.6241***		17.8465***
	Z-bar tilde		5.0275***		11.9606***
HQIC	W-bar	4	7.4847	4	8.2290
	Z-bar		20.2444***		24.5684***
	Z-bar tilde		1.0778		2.0584**

**Fig 8:**
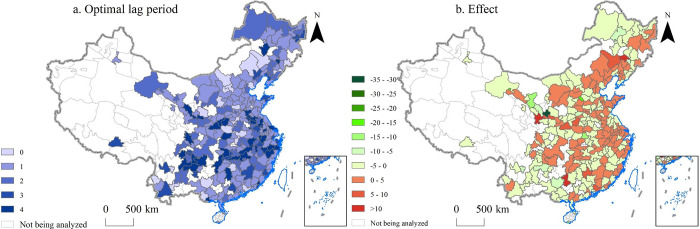
The optimal lag period and effect of average export similarity index (AESI) on market competitive index (MCI). The optimal lag period is the lag period with the minimum mean standard error. Meaning of effect: If AESI is increased by *one* unit, MCI will increase/decrease by the corresponding amount. MCI’s increases are expressed as positive values and decreases as negative values.

The optimal lag periods of 90.37% cities are all greater than 1 (Fig 8A). For more than half of the cities (56.67%), an increase in their AESI led to a decrease in MCI (the light yellow and green areas in Fig 8B). For less than half of the cities (43.33%), an increase in their AESI led to an increase in MCI (the orange and red areas in Fig 8B). These results suggest that there does not appear to be a dominant effect of the impact of AESI on MCI. According to our analysis, this is probably related to the economic level of the city (Appendix C in S1 Appendix). Many cities with negative effects were in the southwest and northeast regions of China, including Kunming City (the capital of Yunnan Province) and Changchun City (the capital of Jilin Province). Many cities with positive effects were in the eastern and central regions of China, including more developed cities, such as Shanghai City and Nanjing City (the capital of Jiangsu Province). This phenomenon shows that more developed cities increase their export share to existing target markets when facing increased competitive pressures.

## Conclusions

Since the beginning of this century, the types of commodities that Chinese cities export to the world have become increasingly diversified, and the number of their target markets has also increased. The development characteristics of these two aspects show that the export trade strength of Chinese cities has increased significantly. Export trade has promoted China’s economic growth and transformation [74, 75]. However, because of the differences in resource endowments, location conditions, and policy treatments of cities in different regions, cities in western, northwestern, and northeastern China did not perform as well as those in southeastern China in terms of diversification of export commodities and export markets.

Based on the ESI, for the AESI that we constructed allows us to analyze the competitive pressure that each city faces. Moreover, AESI has become an important starting point for studying the factors influencing export diversification. From 2000 to 2017, export similarity between Chinese cities increased, and the competition for the global market became fierce. This process was accompanied by a continuous improvement in export diversification. We used Geodetector to discuss the spatial interaction between AESI and CCI/MCI. Notably, AESI explains the spatial pattern of CCI well, but fails to give the same ideal explanation for the spatial pattern of MCI. This stems from the calculation of spatial stratification heterogeneity, which uses cross-sectional data and does not fully incorporate the time element. Fortunately, the PGCT and ALM provide more comprehensive results. The results of the PGCT confirmed the existence of a causal relation between competition and diversification, thus laying a credible foundation for our final effect analysis using ALM.

Through ALM, we have found that competition brings diversification of export commodities. However, the impact of competition on the diversification of export markets has not shown a relatively uniform trend. Moreover, the more backward Chinese cities are, the greater the pressure of competition urges them to increase the variety of goods. This feature is more prominent in the impact of competition on market diversification. Generally, relatively backward cities have a fairly single type of export commodity and a more concentrated target market. Therefore, when the pressure of competition increases, they can use the industrial transfer and development experience of developed cities to broaden their sales channels. By contrast, developed cities have more mature and stable export industrial systems. Hence, when competitive pressure increases, they focus on superior products and key markets, thereby realizing larger-scale economic benefits. Furthermore, the product allocation behavior of an enterprise is an important mechanism for an enterprise to cope with the competition in the export market and thereby improve the productivity of the enterprise [76].

This article reveals an important discovery in the development of China’s urban trade that is, competition is not bad. In the long run, the existence of competition increases the diversification of export commodities of most cities. This is closely related to the anti-risk ability of a city’s trading system, especially for those with more fragile economic structures. COVID-19 has severely affected the export levels of cities in all countries, including China. Resistance to similar crises cannot lack a strong export structure, including the commodity and geographic structures. However, care must also be taken to avoid over-expansion caused by competition. If a city blindly expands its product range or develops a trade market with a low profit margin in order to meet the challenge of competitors, it is highly likely to backfire. On the other hand, the impact of competition on diversification has different results in different cities, and the central government should fully consider the actual situation in different regions when formulating national trade policies in order to achieve improved export performance. Specifically, for those relatively backward cities in the central and western regions that face competitive pressures and choose to expand commodity types and markets, the government should use more policy tools to enable their enterprises to expand overseas markets as far as possible. In fact, with the increasingly smooth trade routes between China, Central Asia, and Europe, the development of foreign trade in inland cities in the central and western regions offers new opportunities.

This study does not include other factors that may affect the diversification of urban exports. If more data can be gathered and sorted in the future, some other economic factors can be taken as control variables. Then, the influence of competition on diversification can be discussed more comprehensively.

## Supporting information

S1 Appendix (DOCX)

S1 Data (RAR)
