# Supplementary material for: Competition among cities for export trade brings diversification: The experience of China’s urban export trade development
Source: PLoS One. 2022 Sep 15;17(9):e0271239. doi: 10.1371/journal.pone.0271239 (PMC9477325; doi:10.1371/journal.pone.0271239)
Supplement: S1 Appendix — (DOCX) [file pone.0271239.s001.docx]

**S1 Appendix.**

**Appendix A.**


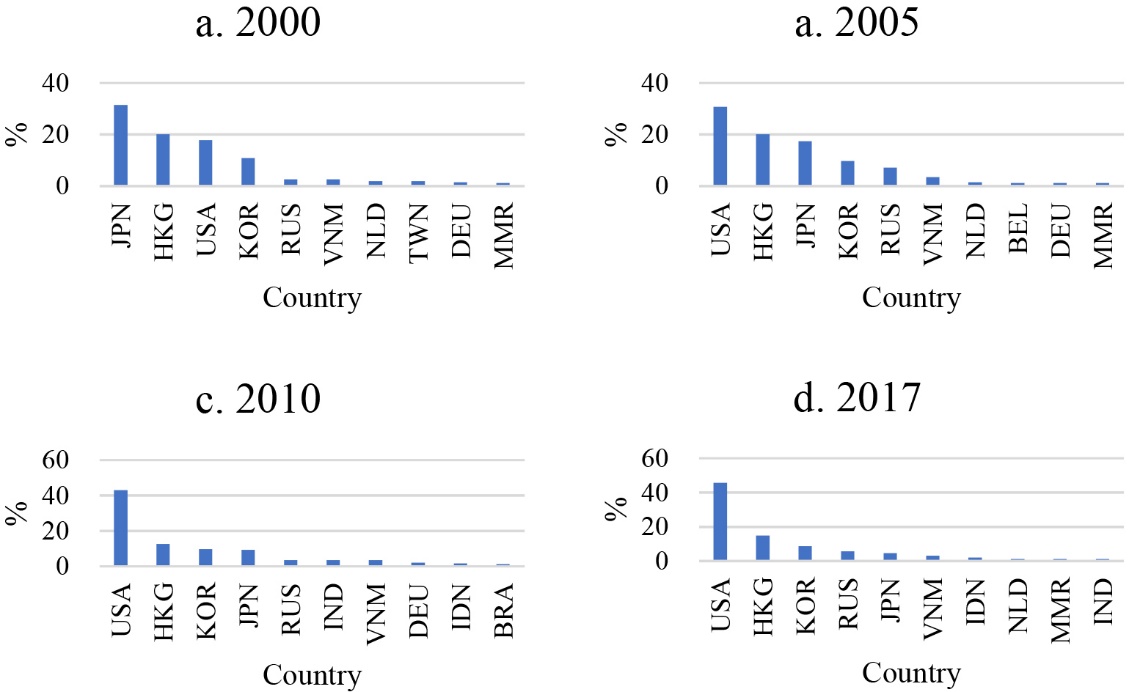


**Fig 1. Proportion of cities that choose different countries as the number one export destination**

**Appendix B.**

The intensity of competition among 11 cities in Jiangxi province is notoriously higher than that among cities in other provinces. In 2017, the AESI of Ji 'an, Fuzhou, and Pingxiang cities in Jiangxi province was 18.20, 17.56, and 17.34, respectively, ranking as the top three in China. Guangzhou and Shenzhen in Guangdong province also had a high AESI of 15.87 and 15.84, respectively. The figure below compares the two provinces. ESI of Ji'an and other cities in Jiangxi, ESI of Fuzhou and other cities in Jiangxi, and ESI of Pingxiang and other cities in Jiangxi were high in 2017 (most ESI were above 40). However, most ESI of Guangzhou/Shenzhen and other cities in Guangdong were below 30. The export products of Jiangxi's cities are very similar, which increases competition among these cities. Take Ji’an as an example, toys (HS1996=850390) were the most exported goods in 2017, accounting for 6.31% of the total value of exports in Ji’an. In the same year, toys were also the most valuable commodity exported by Fuzhou, accounting for 6.69% of the city’s total export value.


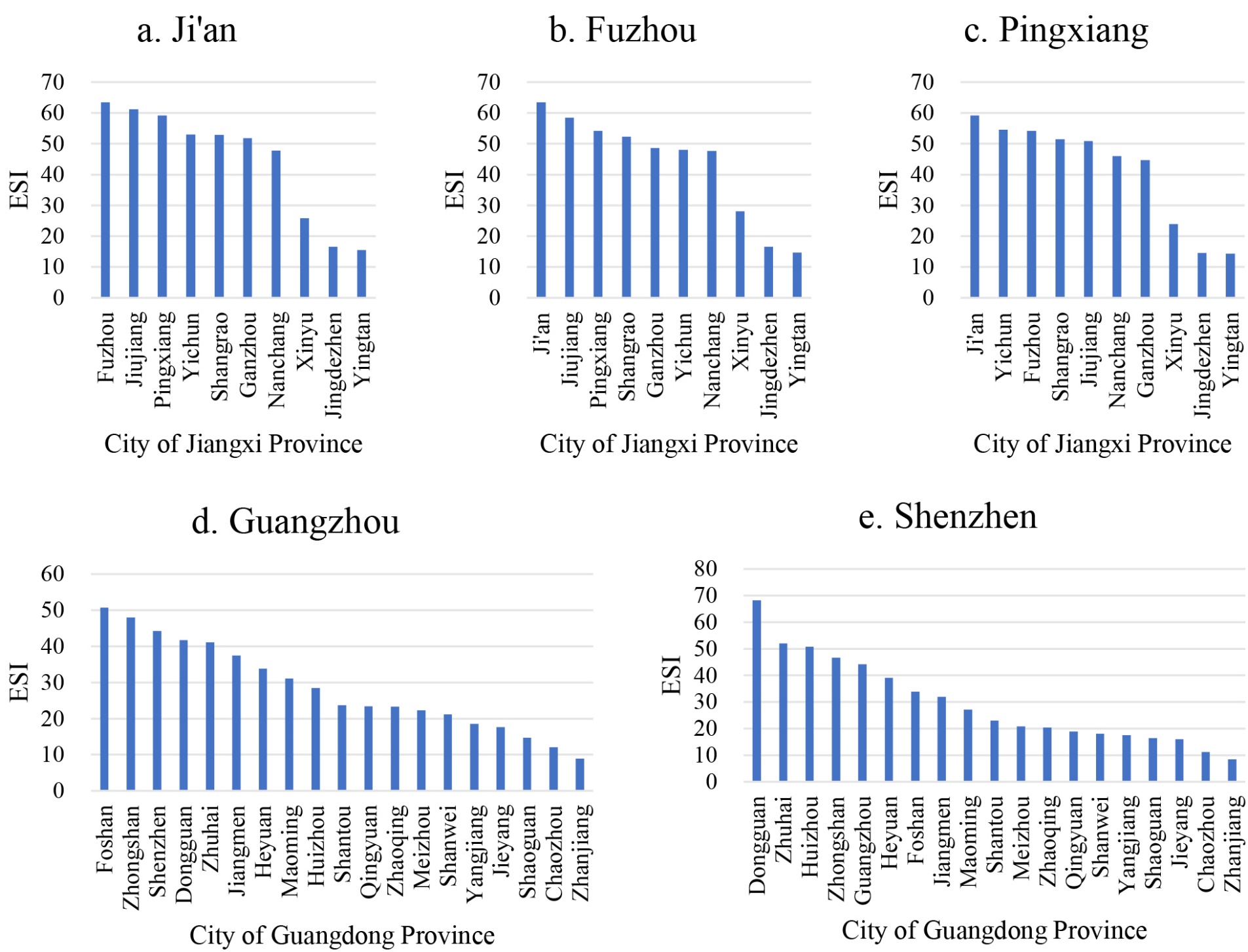


**Fig 2. ESI of Ji’an and other cities, Fuzhou and other cities, Pingxiang and other cities, Guangzhou and other cities, Shenzhen and other cities in 2017**

**Appendix C.**

We divided the average annual GDP of 270 cities into nine groups of 30 cities in descending order. The abscissa of “9” indicates that the 30 cities in this group have the highest annual GDP. The abscissa of “1” indicates that the 30 cities in this group have the lowest annual GDP. In the group with higher annual GDP, the increase of AESI in more cities leads to the increase of MCI. In the group with lower annual GDP, the increase of AESI leads to the decrease of MCI in more cities.


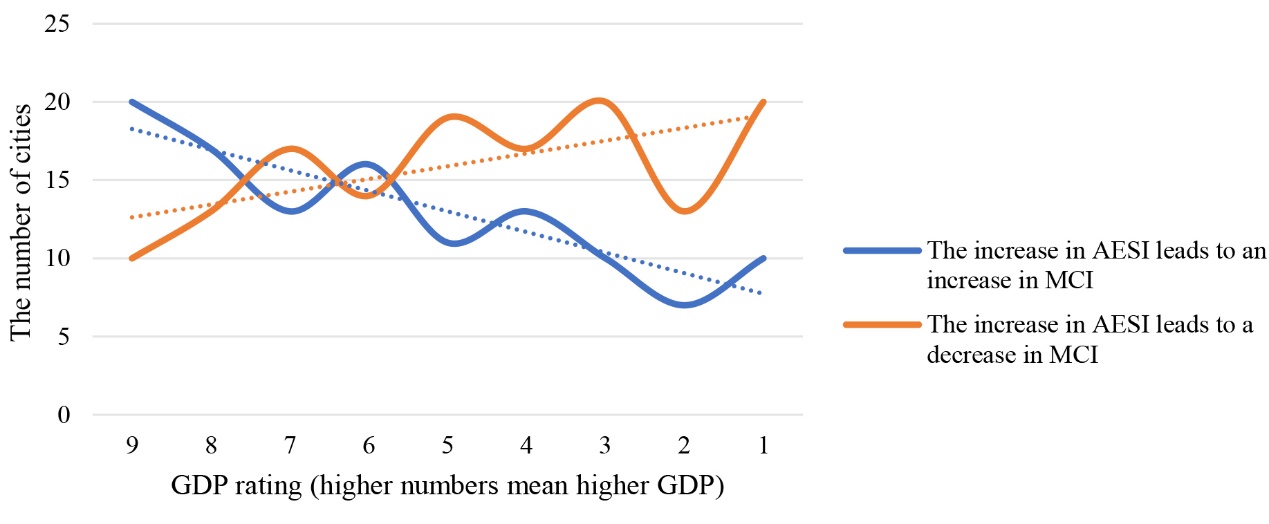


**Fig 3. The effect of AESI on MCI of cities in different GDP groups**
